# The Association between Threatened Miscarriage and Autism Spectrum Disorder and Attention-Deficit/Hyperactivity Disorder in Offspring by Age 14 Years

**DOI:** 10.1007/s10803-024-06251-3

**Published:** 2024-01-28

**Authors:** Daire Buckley, Ali S. Khashan, Fergus P. McCarthy, Karen O’Connor, Gillian M. Maher

**Affiliations:** 1 INFANT Research Centre, University College Cork, Cork, Ireland; 2 School of Public Health, University College Cork, Cork, Ireland; 3 Department of Obstetrics and Gynaecology, University College Cork, Cork, Ireland; 4 Early Intervention in Psychosis Team, RISE, South Lee Mental Health Services, Cork, Ireland; 5 Department of Psychiatry and Neurobehavioral Science, University College Cork, Cork, Ireland

**Keywords:** Autism spectrum disorder, attention-deficit hyperactivity disorder, Threatened miscarriage, Millennium cohort study

## Abstract

**Objective:**

To examine the association between threatened miscarriage, and neurodevelopmental disorders, including autism spectrum disorder (ASD) and attention-deficit/hyperactivity disorder (ADHD) in offspring by age 14 years.

**Methods:**

We used data from the Millennium Cohort Study, a nationally representative longitudinal study of children born in the UK. Data on threatened miscarriage and potential confounders were maternal-reported and collected at 9 months postpartum. Data on ASD and ADHD were based on maternal-reported doctor diagnoses and collected when children were aged 5, 7, 11 and 14 years. A diagnosis of ASD or ADHD was assumed if parents reported ASD or ADHD at age 5, 7, 11 or 14 years. Crude and adjusted logistic regression examined threatened miscarriage and ASD and ADHD relationship, adjusting for several sociodemographic, maternal and lifestyle factors.

**Results:**

A total of 18,294 singleton babies were included at baseline, and 1,104 (6.0%) women experienced a threatened miscarriage during their pregnancy. Adjusted results suggested an association between threatened miscarriage and ASD (OR: 1.55, 95% CI 1.15, 2.08), and ADHD (OR: 1.51, 95% CI 1.09, 2.10) by age 14 years. E-values for threatened miscarriage and ASD were 2.47, while the lower limits of the 95% CI were 1.57. E-values for threatened miscarriage and ADHD were 2.39, while the corresponding lower limits of the 95% CI were 1.40.

**Conclusion:**

Threatened miscarriage was associated with an increased likelihood of ASD and ADHD by the age of 14 years, however, residual confounding cannot be ruled out. Placental pathology may be a potential mechanism for the observed associations.

## Introduction

Vaginal bleeding without cervix dilation within 20 weeks of gestation of a viable intrauterine pregnancy is a symptom of threatened miscarriage. It is a common complication during the first trimester that affects up to 20% of all pregnancies. (Saraswat et al., 2010; Sharma et al., 2020; Dudukina et al., 2021) Maternal risk factors for threatened miscarriage include maternal overweight and obesity, cigarette smoking, and alcohol consumption, lack of physical exercise or stress, advanced age, and previous history of miscarriages. (Wahabi et al., 2018; Dudukina et al., 2021) Evidence suggests that vaginal bleeding is associated with an increased risk of multiple adverse antenatal complications such as preeclampsia, preterm birth, and intrauterine growth restriction (IUGR) (Saraswat et al., 2010; Liu et al., 2022; Zhong et al., 2022).

Neurodevelopmental disorders (NDD) may be dependent on the timing of exposure and/or genetic predisposition to certain environmental stimuli before birth, yet the exact aetiology of NDDs such as autism spectrum disorder (ASD) and attention-deficit/hyperactivity disorder (ADHD) are complex and poorly understood. (Glasson et al., 2004; Kolevzon et al., 2007; Autism Spectrum Disorder, 2018; Varcin et al., 2019) Previous studies have investigated whether complications that arise during the pre- and perinatal periods, can predispose a foetus’s vulnerability to developing NDDs (Wang et al., 2017). The findings report increases in the number of obstetric complications linking factors such as advanced maternal/paternal age, maternal pre-pregnancy obesity, preeclampsia, and extremely premature birth (prior to 26 weeks gestation) to ASD, while factors such as cigarette and alcohol exposure during pregnancy have been previously linked to ADHD (Gillberg & Gillberg, 1983; Lord et al., 1991; Burd et al., 1999; Reichenberg et al., 2006; Carlsson et al., 2021).

Threatened miscarriage has also been associated with adverse neurodevelopmental outcomes. Since early placentation coincides with organogenesis, including the development of the neural tube, it has been hypothesised that first-trimester bleeding may be an indication of placental dysfunction, which may manifest as a potential mechanism for poor neurodevelopmental outcomes from pregnancies.(Saraswat et al., 2010; Maher et al., 2020; Maher et al., 2019; Emara, 2020; Dudukina et al., 2021; Liu et al., 2022) One 2017 meta-analysis reported that offspring born from a gestation affected by threatened miscarriage have a two- to three-fold increased likelihood of developing ASD.(Wang et al., 2017). Furthermore, a Danish registry-based cohort study estimated that the likelihood of an ADHD diagnosis by age 16 years increased by 30% for children exposed to threatened miscarriage in utero in its unadjusted analysis. However, this result was attenuated in adjusted analysis.(Dudukina et al., 2021).

Evidence examining the association between threatened miscarriage and ASD and ADHD are sometimes inconsistent, potentially due to different methodologies, varying degrees of adjustment for confounding and different ranges in follow-up. (Ahmadvand et al., 2023; Dudukina et al., 2021; Glasson et al., 2004; Say et al., 2016). If observed associations were causal, they may inform the potential need for increased developmental screening of infants exposed to threatened miscarriage in utero to allow early intervention which may improve neurodevelopmental outcome (Zwaigenbaum et al., 2015).

Therefore, the objective of this study was to investigate whether there is an association between threatened miscarriage and the subsequent likelihood of ASD, and ADHD in offspring, by the age of 14 years, using data from a nationally representative longitudinal study of children born in the United Kingdom and adjusting for several potential important confounding factors.

## Methods

### Cohort

The Millennium Cohort Study (MCS) is an ongoing longitudinal birth cohort study, set up to follow the lives of children born between 2000 and 2002 in the United Kingdom. The population was constructed to be a nationally representative sample of the UK population and contains data that was collected from 18,552 families (18,827 children). A cluster sampling design was used, stratified first by region within the UK, i.e., Scotland, England, Wales, and Northern Ireland, then by electoral ward, the ethnic composition of the ward, and social disadvantage. Baseline data was collected when the infants were 9 months, with an additional six survey sweeps completed to date - see **Table S1** in the supplement. Briefly, follow-ups have currently been conducted at ages 3 years (MCS2), 5 years (MCS3), 7 years (MCS4), 11 years (MCS5), 14 years (MCS6) and 17 years (MCS7) (Connelly & Platt, 2014).

### Exposure

Threatened miscarriage was measured during wave one, when the children were nine months old, through a face-to-face computer-assisted personal interview. Mothers were asked if they had suffered “*any illnesses or problems during pregnancy requiring medical attention and treatment*”. If the answer to this question was “*yes*”, they were then instructed to choose all that apply from a list of illnesses. The list included, “*Bleeding or threatened miscarriage in early pregnancy*”. If the answer box to this was ticked “*yes*”, then threatened miscarriage was assumed.

### Outcome

When children were aged 5, 7, 11 and 14 years, parents were asked, in two separate questions, if a doctor or health professional had ever told them that their child had ASD or ADHD. If the respondent answered “*yes”* at any of these time points, then the child was considered to have ASD or ADHD.

### Confounding Variables

In the main analysis, we included only covariates in our model, which we believe to be associated with the exposure and outcome and have excluded any variables that might be potential mediators of the association. Therefore, we controlled for the following potential confounders, all of which were determined *a priori* and based on previous literature. (Curran et al., 2016, 2018; Gallagher et al., 2018; Bohm et al., 2019)

*Maternal age* was measured in years and referred to mothers’ age at the time of birth. *Maternal education* is the highest academic qualification achieved, which was re-categorised, and ranged from diploma or above, A-levels, O-level to less than O-level. *Household income is* categorised according to the Organisation for Economic Co-operation and Development (OECD) income-weighted quintiles. *Maternal smoking status* mothers were asked the number of cigarettes they smoked per day before pregnancy and the number smoked per day if they changed the amount smoked during pregnancy. This information was re-categorised as a non-smoker, quit during pregnancy and smoked during pregnancy. *Maternal alcohol consumption during pregnancy* the frequency of alcohol consumption during pregnancy was categorised as yes/no. *Pre-pregnancy body mass index (BMI)* maternal weight and height prior to pregnancy were self-reported and used to calculate maternal BMI. This variable was then re-categorised as underweight, normal weight, overweight, obese, and unknown. *Depression or mental illness during pregnancy* mothers were asked whether a doctor told them that they suffer from depression or anxiety during their pregnancy, this was categorised as yes/no. *Infant sex* was categorised as male/female. *Parity* was derived from the number of siblings in the household, and re-categorised as either primiparous or multiparous.

### Statistical Analysis

Data were analysed using SPSS version 28.0. Crude and multivariable logistic regression analysis estimated odds ratios (OR) and 95% confidence intervals (95% CI) to examine the association between threatened miscarriage and ASD and ADHD in the offspring. For each outcome, four logistic regression models were performed: Model 1 represented the crude model. Model 2 represented the confounder-adjusted analysis controlling for sociodemographic factors including maternal age, maternal education, and household income. Model 3 represented the confounder-adjusted analysis controlling for maternal and lifestyle including maternal smoking status, maternal alcohol consumption during pregnancy, pre-pregnancy body mass index (BMI), maternal depression or anxiety, infant sex, and parity. Model 4 represented the fully adjusted analysis controlling for both sociodemographic factors in Model 2 and maternal and lifestyle factors in Model 3.

***E-value***: We calculated the E-value for the statistically significant primary effect estimates and lower limits of their 95% confidence interval (CI) to examine the extent of unmeasured confounding, using the publicly available online E-value calculator: https://evalue.hmdc.harvard.edu/app/ (VanderWeele & Ding, 2017; Mathur et al., 2018). In summary, an E-value is a continuous measure that quantifies the minimum strength of association an unmeasured confounder would need to have with both miscarriage threat and ASD, and miscarriage threat and ADHD to explain away an effect estimate. (VanderWeele & Ding, 2017)

***Sensitivity analysis***: As placental complications are more common in males; we stratified the main analyses by sex (categorised as male and female). In addition, we repeated the main analyses examining threatened miscarriage-ASD and threatened miscarriage-ADHD in separate models: first, stratified with and without hypertensive disorders of pregnancy (HDP), second, stratified with and without being born small for gestational age (SGA) and third, stratified with and without being born preterm. HDP was defined as the new onset of raised blood pressure in a previously normotensive individual. During the survey, the question was posed to the participant, *“Did you have any illnesses or other problems during your pregnancy that required medical attention or treatment?”* If the answer to this question was *“yes”*, a list of illnesses was provided, within which they were instructed to choose all that apply. The list included, *“Raised blood pressure, eclampsia/preeclampsia or toxaemia*”. If this box was ticked “*yes”*, then a diagnosis of hypertensive disorders during pregnancy was assumed. SGA was defined as a birth weight of less than the 10th percentile for the gestational age and sex of the child and was based on maternal reporting of birthweight, gestational age, and infant sex. Preterm birth was based on maternal reporting about their child’s gestational age in days. This was converted to weeks and re-categorised as < 37 weeks’ gestation and ≥ 37 weeks’ gestation. In addition to this, we stratified results among those ever diagnosed with maternal depression or anxiety in a separate analysis. Finally, we examined the distribution of the most prevalent reported illnesses or problems during pregnancy that required medical attention and treatment in our exposed and unexposed group using a chi squared test (see **Table S2** in the supplement). We then examined the association between threatened miscarriage and ASD and ADHD excluding those with any significant reported illness. All sensitivity analyses were performed on the fully adjusted model (Model 4).

## Results

A total of 18,294 singleton mother-child pairs were included in wave one of the Millennium Cohort Study. Of these, 1,104 (6.0%) women experienced a threatened miscarriage during pregnancy. 15,945 respondents answered the question of whether a doctor or health professional had ever told them their child had ASD or ADHD by age 14 years. Of these, 575 (3.7%) reported a diagnosis of ASD and 497 (3.2%) reported a diagnosis of ADHD. (Table 1.)

**Table 1:** Mother and Child Characteristics among Millennium Cohort Study Participants

CHARACTERISTICS	No Threatened Miscarriage	Threatened Miscarriage
*Total population, n (%)*	17,190 (94.0)	1104 (6.0)
*Infant sex, n (%)*		
Male	8823 (51.3)	592 (53.6)
Female	8367 (48.7)	512 (46.4)
*Autism Spectrum Disorder (ASD), n (%)*		
No	14,038 (96.4)	882 (94.4)
Yes	523 (3.6)	52 (5.6)
*Attention-Deficit Hyperactivity Disorder (ADHD), n (%)*		
No	14,104 (96.9)	892 (95.5)
Yes	455 (3.1)	42 (4.5)
*Parity, n (%)*		
Nulliparous	7214 (42.0)	479 (43.4)
Multiparous	9976 (58.0)	625 (56.6)
*Maternal age, years, n (%)*		
14–19	1501 (8.7)	83 (7.5)
20–29	8063 (46.9)	501 (45.4)
30–39	7215 (42.0)	497 (45.0)
40–99	360 (2.1)	23 (2.1)
Unknown/Missing	51 (0.3)	0 (0.0)
*Maternal Education, Completed, n (%)*		
Less than O-level	3383 (19.7)	174 (15.8)
O-level	7541 (43.9)	519 (47.0)
A-level	1591 (9.3)	105 (9.5)
Diploma or above	4100 (23.9)	285 (25.8)
Unknown/Missing	575 (3.3)	21 (1.9)
*Maternal smoking status, n (%)*		
Non-smoker	11,008 (64.0)	715 (64.8)
Quit during pregnancy	2170 (12.6)	149 (13.5)
Smoked during pregnancy	3985 (23.2)	240 (21.7)
Unknown/Missing		
*Maternal alcohol consumption during pregnancy, n (%)*		
No	12,213 (71.0)	776 (70.2)
Yes	4921 (28.6)	328 (29.7)
Unknown/Missing	56 (0.3)	0 (0.0)
*Maternal pre-pregnancy BMI, n (%)*		
Underweight (≤ 18.5 kg/m^2^)	943 (5.5)	65 (5.9)
Normal weight (18.5–24.9 kg/m^2^)	10,160 (59.1)	659 (59.7)
Overweight (25–29.9 kg/m^2^)	3134 (18.2)	211 (19.1)
Obese (30–39 kg/m^2^)	1347 (7.8)	97 (8.8)
Unknown/Missing	1606 (9.3)	72 (6.5)
*Household income, n (%)*		
Lowest quintile	4340 (25.2)	240 (21.7)
2nd quintile	3881 (22.6)	221 (20.0)
3rd quintile	3227 (18.8)	223 (20.2)
4th quintile	2943 (17.1)	228 (20.7)
Highest quintile	2720 (15.8)	189 (17.1)
Unknown/Missing	79 (0.5)	3 (0.3)
*Maternal depression or anxiety (ever diagnosed), n (%)*		
No	13,050 (75.9)	729 (66.0)
Yes	4115 (23.9)	375 (34.0)

Data refer to the number (percentage), n (%) or mean and standard deviation (SD) where appropriate.

Abbreviations: BMI, body mass index

### Logistic Regression

#### Threatened Miscarriage and ASD

In the crude analysis, Model 1, the odds ratio (OR) of the association between threatened miscarriage and ASD was 1.58 [95% CI 1.18 to 2.12]. This association remained statistically significant in the fully adjusted model (Model 4) (OR 1.54 [95% CI 1.14 to 2.07]), suggesting that threatened miscarriage was associated with a 54% increase in odds of an ASD diagnosis by age 14. (Table 2).

#### Threatened Miscarriage and ADHD

In an unadjusted analysis for ADHD, Model 1, the relationship between threatened miscarriage and ADHD was statistically significant, OR 1.46 [95% CI 1.06 to 2.02]. The odds increased the estimated association slightly after adjusting for confounders in Model 4 (OR 1.51 [1.09 to 2.10]) suggesting threatened miscarriage was associated with a 51% increase in the odds of an ADHD diagnosis by age 14. (Table 2.)

**Table 2:** Logistic Regression for the association between Threatened Miscarriage and ASD and ADHD in the offspring by age 14 years, among Millennium Cohort Participants

ASD	No. of exposed cases	Model 1 OR [95% CI]	Model 2 OR [95% CI]	Model 3 OR [95% CI]	Model 4 OR [95% CI]
*Threatened miscarriage*	52	1.58 [1.18 to 2.12]	1.61 [1.20 to 2.16]	1.53 [1.14 to 2.06]	1.55 [1.15 to 2.08]
ADHD	**No. of exposed cases**	**Model 1** **OR [95% CI]**	**Model 2** **OR [95% CI]**	**Model 3** **OR [95% CI]**	**Model 4** **OR [95% CI]**
*Threatened miscarriage*	42	1.46 [1.06 to 2.02]	1.56 [1.13 to 2.16]	1.46 [1.05 to 2.02]	1.51 [1.09 to 2.10])

Model 1: Crude analysis

Model 2: Adjusted for maternal age, maternal education, household income

Model 3: Adjusted for maternal smoking status, maternal alcohol consumption during pregnancy, pre-pregnancy BMI, maternal depression during pregnancy, infant sex, and parity.

Model 4: fully adjusted model

Abbreviations: ASD, autism spectrum disorder; ADHD, attention-deficit hyperactivity disorder; OR, odds ratio; 95% CI, 95% confidence interval [lower to upper]; BMI, body mass index

### E-Values

#### Threatened Miscarriage and ASD

The E-values for significant primary effect estimates were 2.47, while the E-values for corresponding lower limits of the 95% CI were 1.57. (See **Table S3** in the supplement for worked examples of threatened miscarriage and ASD).

#### Threatened Miscarriage and ADHD

The E-values for significant primary effect estimates were 2.39, while the E-values for corresponding lower limits of the 95% CI were 1.40. (See **Table S4** in the supplement for worked examples of threatened miscarriage and ADHD).

### Sensitivity Analysis

**Threatened miscarriage and ASD**: Among male infants, the OR for the association between threatened miscarriage and ASD was 1.55 [95% CI: 1.11 to 2.18]; p-value for interaction: 0.010. While the OR was similar for females, the result was not statistically significant, potentially due to the smaller number of exposed cases compared to males (OR: 1.51 [95% CI: 0.80 to 2.83]; p-value for interaction: 0.198).

Excluding HDP did not materially change results (OR: 1.41 [95% CI: 1.01 to 1.97]; p-value for interaction: 0.043), while the OR for threatened miscarriage-ASD among those exposed to HDP increased to 2.01 [95% CI: 1.03 to 3.92]; p-value for interaction: 0.040 and maintained statistical significance. Similarly, the results of sensitivity analysis excluding those born SGA were similar to our main findings, while the OR for the association between threatened miscarriage and ASD among those born SGA was 2.17 [95% CI 0.82 to 5.74], with the p-value for interaction: 0.115.

The exclusion of preterm births led to a slight increase in the relationship between threatened miscarriage and ASD (OR 1.62 [95% CI 1.18 to 2.22], p-value of interaction: 0.003), while we found no association between threatened miscarriage and ASD among those born preterm (OR: 0.99 [95% CI: 0.41 to 2.40]) Exclusion of women ever diagnosed with maternal depression or anxiety led to a slight reduction in OR (OR 1.37 [95% CI 0.92 to 2.06] p-value for interaction: 0.966), which was no longer statistically significant, while the OR increased to 1.65 [95% CI: 1.04 to 2.57], the p-value for interaction: 0.027, among those ever diagnosed with maternal depression or anxiety. Finally, excluding those who reported bleeding in later pregnancy, persistent vomiting, raised blood pressure/preeclampsia, urinary infection, and non-trivial infections during pregnancy did not materially change results, however results were no longer statistically significant (OR: 1.32 [0.85 to 2.07]) (Table 3).

**Table 3:** Sensitivity analysis examining the association between Threatened Miscarriage and ASD and ADHD in the offspring by age 14 years, among Millennium Cohort Study Participants

	No. of exposed cases	OR [95% CI] ^*^	p for interaction	No. of exposed cases	OR [95% CI] ^*^	p for interaction
ASD	**Males**			**Females**		
*Threatened miscarriage*	41	1.55 [1.11 to 2.18]	0.010	11	1.51 [0.80 to 2.83]	0.198
	**No HDP**			**with HDP**		
*Threatened miscarriage*	40	1.41 [1.01 to 1.97]	0.043	12	2.01 [1.03 to 3.92]	0.040
	**Not SGA**			**SGA**		
*Threatened miscarriage*	47	1.49 [1.09 to 2.03]	0.012	5	2.17 [0.82 to 5.74]	0.115
	**No PTB**			**PTB**		
*Threatened miscarriage*	46	1.62 [1.18 to 2.22]	0.003	6	0.98 [0.41 to 2.36]	0.966
	**No maternal depression/anxiety**			**With maternal depression/anxiety**		
*Threatened miscarriage*	27	1.37 [0.92 to 2.06]	0.130	25	1.65 [1.06 to 2.57]	0.027
	**Excluding additional illnesses during pregnancy** ^**^					
*Threatened miscarriage*	22	1.32 [0.85 to 2.07]	-	-	-	-
ADHD		**Males**		**Females**		
*Threatened miscarriage*	38	1.74 [1.22 to 2.48]	0.002	4	0.69 [0.25 to 1.89]	0.470
	**No HDP**			**With HDP**		
*Threatened miscarriage*	34	1.42 [0.98 to 2.04]	0.058	8	1.97 [0.88 to 4.43]	0.100
	**Not SGA**			**SGA**		
*Threatened miscarriage*	39	1.54 [1.09 to 2.18]	0.013	3	1.16 [0.34 to 3.89]	0.809
	**No PTB**			**PTB**		
*Threatened miscarriage*	35	1.47 [1.03 to 2.11]	0.033	7	1.62 [0.69 to 3.79]	0.264
	**No maternal depression/anxiety**			**With maternal depression/anxiety**		
*Threatened miscarriage*	25	1.66 [1.09 to 2.54]	0.018	17	1.20 [0.72 to 2.03]	0.478
	**Excluding additional illnesses during pregnancy** ^**^					
*Threatened miscarriage*	19	1.38 [0.85 to 2.22]	-	-	-	-

*Models are fully adjusted for maternal age, maternal education, household income, maternal smoking status, maternal alcohol consumption during pregnancy, pre-pregnancy BMI, maternal depression during pregnancy, infant sex, and parity.

**Bleeding in later pregnancy, persistent vomiting, raised blood pressure/preeclampsia, urinary infection, and non-trivial infections.

Abbreviations: OR, odds ratio; 95% CI, 95% confidence interval; ASD, autism spectrum disorder; ADHD, attention-deficit hyperactivity disorder; HDP, hypertensive disorder; SGA, small-for-gestational-age; PTB, preterm birth

Threatened miscarriage and ADHD: Among male infants, the OR for the association between threatened miscarriage and ADHD was 1.74 [1.22 to 2.48], and the p-value for interaction was 0.002. While the OR for females changed materially, the result was not statistically significant (OR 0.69 [0.25 to 1.89], the p-value for interaction: 0.470. Excluding HDP led to a reduction in the observed relationship between threatened miscarriage-ADHD, OR 1.42 [95% CI: 0.98 to 2.04]; p-value for interaction: 0.058) which was no longer statistically significant, while the OR among those exposed to HDP increased to 1.97 [95% CI: 0.88 to 4.43], p-value for interaction: 0.100, however, this also did not reach statistical significance. Similarly, the results of sensitivity analysis excluding those born SGA were similar to our main findings, while the OR for the association between threatened miscarriage and ADHD among those born SGA was reduced to 1.16 [95% CI: 0.34 to 3.89], the p-value for interaction: 0.809. The exclusion of preterm births led to a minor decrease in the relationship between threatened miscarriage and ADHD (OR 1.45 [95% CI 1.01 to 2.07]), while we found an increase in the relationship between threatened miscarriage and ADHD among those born preterm (OR: 1.85 [95% CI: 0.76 to 4.49]), again, this was no longer classified as statistically significant. The exclusion of women ever diagnosed with maternal depression or anxiety led to a slight increase in OR 1.66 [95% CI 1.09 to 2.54], p-value for interaction: 0.018), while the OR reduced among those ever diagnosed with maternal depression or anxiety to 1.20 [95% CI: 0.72 to 2.03] p-value for interaction: 0.478, which was no longer statistically significant. Finally, similar to above, excluding those who reported bleeding in later pregnancy, persistent vomiting, raised blood pressure/preeclampsia, urinary infection, and non-trivial infections during pregnancy did not materially change results (OR: 1.38 [0.85 to 2.22]) (Table 3).

## Discussion

### Main Findings

In this retrospective population-based cohort study, we examined the association between threatened miscarriage and ASD and ADHD by age 14 years. We have yielded two principal findings. First, threatened miscarriage was associated with a 55% increase in the likelihood of ASD. Second, threatened miscarriage was associated with a 51% increase in the likelihood of ADHD. These associations remained significant after controlling for several potential confounders including maternal age, maternal education, household income, maternal smoking status, maternal alcohol consumption during pregnancy, pre-pregnancy body mass index, depression or mental illness during pregnancy, infant sex, and parity.

In agreement with this data, other published studies (Glasson et al., 2004; Kolevzon et al., 2007; Autism Spectrum Disorder, 2018) support these findings, reporting that haemorrhage without cervix dilation within the first 20 weeks of gestation is a commonly reported symptom among ASD/ADHD case mothers.

Furthermore, congruent with other research, our findings observed a significant association between threatened miscarriage and ASD and ADHD among male infants. (*Centers for Disease Control Autism and Developmental Disabilities Monitoring (ADDM) Network*, 2018; Carlsson et al., 2021) While the association between threatened miscarriage and ASD among females was not statistically significant for ASD, and reduced odds were demonstrated for ADHD, some studies theorise that NDDs in females might be underdiagnosed, potentially biasing the results towards the null. (Carlsson et al., 2021). Furthermore, we found some evidence exposure to threatened miscarriage and being born SGA was associated with an increased likelihood of an ASD diagnosis. Both vaginal bleeding and SGA are associated with placental dysfunction. (Saraswat et al., 2010; Miller et al., 2016; Maher et al., 2019) Placental dysfunction impacts the adequate supply of nutrients and oxygen provided to the baby and the removal of harmful waste from the baby. As the central organ responsible for the regulation of these maternal and foetal interactions, a compromised placenta fails to properly support the developing foetus and is therefore hypothesised to be a cause of adverse neurodevelopmental consequences for a developing brain. (Miller et al., 2016).

### Strengths and Limitations

The current study has several strengths. First, we used data from a large, nationally representative cohort of children born in the UK between 2000 and 2002. Second, this cohort contains access to a wide variety of information including economic, sociodemographic, and perinatal variables which have been inconsistently accounted for in previous studies. (Saraswat et al., 2010; Wang et al., 2017). This allowed for the adjustment of many potential confounders such as maternal age, maternal education, household income, maternal smoking status, maternal alcohol consumption during pregnancy, pre-pregnancy body mass index, depression or mental illness during pregnancy, infant sex, and parity. Further, the data allowed us to conduct sensitivity analyses for variables that could be considered as mediators or potential confounders, by stratifying for those with and without hypertensive disorders of pregnancy (HDP), those with and without being born small for gestational age (SGA), and born preterm, we also stratified the results among those ever diagnosed with maternal depression or anxiety.

The study also has some limitations that should be noted. First, data on potential confounders were self-reported at nine months postpartum and therefore subject to recall bias. However, the agreement between self-reported lifestyle factors such as smoking pre-pregnancy and during pregnancy with antenatal records has been shown to be good very good 4–9 years post-delivery.(Rice et al., 2007; Skulstad et al., 2017). Additionally, as data on potential confounders were collected 9 months post-delivery in the current study, it is likely to be more accurate than data collected 4–9 years after pregnancy. Second, threatened miscarriage was also subject to recall bias. However, when measuring our exposure, mothers were asked if their illness or problem during pregnancy required medical attention and treatment. The requirement for medical attention/treatment may improve recall as the event is likely more salient to the individual. Furthermore, the rate of exposure observed in the current study (6.0%) is somewhat comparable to that reported in a Danish registry-based cohort which used ICD-coding to classify threatened miscarriage (Dudukina et al., 2021). Nevertheless, the rate of exposure observed in the current study (6.0%) may be underestimated compared to previously reported estimates of threatened miscarriage of up to 20% (Sharma et al., 2020). However, any misclassification of the exposure is likely to be non-differential and therefore potentially biasing results towards the null.

Third, reporting of ASD and ADHD within this cohort is also based solely on parental recall which could potentially introduce misclassification of our outcomes. (Kolevzon et al., 2007). However, it is not uncommon to see parent-reported neurodevelopmental disorders being used in previously published research. (Rice et al., 2007; Blumberg et al., 2013; Russell et al., 2014; Curran et al., 2016; Skulstad et al., 2017) Furthermore, as parents were unaware of the current study hypothesis at the time of data collection, any misclassification would likely be non-differential.

Fourth, the case assessment diagnosis for both neurodevelopmental disorders in this study did not include confirmation from the gold standard measurement tools such as the Autism Diagnostic Observation Schedule (ADOS) or the Autism Diagnostic Interview-Revised (ADI-R). However, the prevalence of ASD within this cohort (3.7%) is comparable to that reported in English primary care data for 2020–2021 (O’Nions et al., 2023) and is somewhat comparable to recent data figures published by the Centers for Disease Control Autism and Developmental Disabilities Monitoring (ADDM) Network, 2020. Likewise, the prevalence of parent-reported ADHD within this cohort (3.2%) is similar to UK-rates reported elsewhere (National Health Service, 2018), however it is lower compared to estimates reported in the United States. (Russell et al., 2014; Centers for Disease Control and Prevention Attention-Deficit/Hyperactivity Disorder (ADHD), 2019). This discrepancy could be due to differing diagnostic criteria and cultural practices when considering the label of ADHD or, albeit less likely, due to truly lower levels of ADHD symptoms in the UK. (Russell et al., 2014). Fifth, there is a lack of data on family history of ASD and ADHD diagnosis, and genetic sequencing within the participants of the cohort is not available, therefore, these variables could not be controlled for as confounding factors within the analyses. It has been established from previous studies and in clinical settings, that ASD and ADHD are high heritability disorders owing to inherited genetic influences, such that, ASD is estimated at 80% and ADHD at 74%, respectively. (Bai et al., 2019; Faraone & Larsson, 2019). Therefore, unmeasured confounding, including confounding due to shared genetics, cannot be ruled out. However, the relatively high E-values suggest the observed associations may be less likely to be explained away by unmeasured confounding factors. Finally, loss to follow-up may have introduced selection bias in the current study. For example, our sample size included 18,294 singleton mother-child pairs at wave 1. This was reduced to 15,590 in wave 2, 15,246 in wave 3, 13,857 in wave 4, 13, 287 in wave 5, and 11,726 in wave 6. As participation in follow-up research can be influenced by several factors, and children with behavioural disorders are more likely to be lost to follow-up, this may have biased our results towards the null (Wolke et al., 2009).

## Conclusion

Threatened miscarriage was associated with an increased likelihood of ASD and ADHD by age 14 years. However, it is not possible to rule out the presence of residual or unmeasured confounding including familial confounding due to shared genetics. Placental pathology may be a potential mechanism for the observed associations.
